# Structural and functional characterization of the receptor binding proteins of *Escherichia coli* O157 phages EP75 and EP335

**DOI:** 10.1016/j.csbj.2021.06.001

**Published:** 2021-06-04

**Authors:** Sander Witte, Léa V. Zinsli, Rafael Gonzalez-Serrano, Cassandra I. Matter, Martin J. Loessner, Joël T. van Mierlo, Matthew Dunne

**Affiliations:** a Micreos Food Safety B.V., Wageningen, Nieuwe Kanaal 7P, 6709PA, The Netherlands; b Institute of Food Nutrition and Health, ETH Zürich, Schmelzbergstrasse 7, 8092 Zürich, Switzerland; c Evolutionary Genomics Group, Universidad Miguel Hernández, San Juan de Alicante, Spain

**Keywords:** Bacteriophage, STEC, *Escherichia coli* O157, *Salmonella*, Tail fiber, Tailspike, Receptor binding protein, Lipopolysaccharide, O-antigen

## Abstract

Bacteriophages (phages) are widely used as biocontrol agents in food and as antibacterial agents for treatment of food production plant surfaces. An important feature of such phages is broad infectivity towards a given pathogenic species. Phages attach to the surfaces of bacterial cells using receptor binding proteins (RBPs), namely tail fibers or tailspikes (TSPs). The binding range of RBPs is the primary determinant of phage host range and infectivity, and therefore dictates a phage’s suitability as an antibacterial agent. Phages EP75 and EP335 broadly infect strains of *E. coli* serotype O157. To better understand host recognition by both phages, here we focused on characterizing the structures and functions of their RBPs. We identified two distinct tail fibers in the genome of the podovirus EP335: gp12 and gp13. Using fluorescence microscopy, we reveal how gp13 recognizes strains of *E. coli* serotypes O157 and O26. Phage EP75 belongs to the *Kuttervirus* genus within the *Ackermannviridae* family and features a four TSP complex (TSPs 1–4) that is universal among such phages. We demonstrate enzymatic activity of TSP1 (gp167) and TSP2 (gp168) toward the O18A and O157 O-antigens of *E. coli,* respectively, as well as TSP3 activity (gp169.1) against O4, O7, and O9 *Salmonella* O-antigens. TSPs of EP75 present high similarity to TSPs from *E. coli* phages CBA120 (TSP2) and HK620 (TSP1) and *Salmonella* myovirus Det7 (TSP3), which helps explain the cross-genus infectivity observed for EP75.

## Highlights

• Receptor binding proteins (RBPs) are the primary determinants of phage host range.
• Tail fiber of phage EP335 (gp13) recognizes *E. coli* serotypes O157 and O26.
• Phage EP75 tailspikes are active towards *E. coli* and *Salmonella* O-antigens.
• Cross-genus tailspike activity is consistent with the broad host range of phage EP75.

## Graphical abstract

**Figure:**
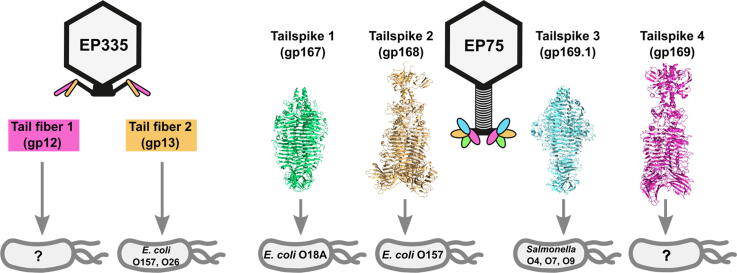

## 1 Introduction

Despite the strict hygiene standards applied during food production and processing in the Western world, foodborne diseases remain a considerable burden on global human health [1], [2]. In the US alone, around 48 million people acquire a foodborne illness annually resulting in 128,000 hospitalizations and 3,000 deaths [3]. An important group of foodborne pathogens are Shiga toxin-producing *Escherichia coli* (STEC), illustrated by major STEC outbreaks in Europe [4] and the US, e.g., linked to romaine lettuce [5], [6], and frequently caused by *E. coli* O157 [7]. Typically, a STEC infection results in common gastroenteritis and can lead to the development of life-threatening hemolytic uremic syndrome (HUS) [8]. Data obtained in 2019 by the Foodborne Diseases Active Surveillance Network (FoodNet) showed that the incidence of laboratory-diagnosed STEC infections in the US continues to rise [5]. This implies a lack of progress in the global control of STEC contaminations during food production, and thus calls for the implementation of new biocontrol strategies.

Bacteriophages (phages) are viruses that infect and kill specific host bacteria, while leaving human cells and non-target bacteria unaffected [9]. The unique ability of phages to target bacteria with species- or even strain-level specificity is a major force behind the resurgence of interest in phage-based therapeutics (e.g., phage therapy), especially for treating antibiotic resistant bacterial infections [10], [11]. In addition to their therapeutic potential, phages are increasingly used as preservatives in a wide variety of food products and as antibacterial agents in food production facilities [12]. Currently there are 12 commercially available phage-based products that have been granted Generally Recognized as Safe (GRAS) status by the Food and Drug Administration (FDA) and are being used to preserve post-harvest foods, meat, poultry, and egg products [12], [13]. Phages must meet certain requirements to be considered safe as a food preservative; for example, they must be of natural origin, non-genetically modified, incapable of genetic transduction, and strictly lytic (i.e., lack the ability to integrate as a prophage). Importantly, phages must have a broad host range to ensure wide spectrum antibacterial activity against different strains of a target pathogen. In the case of *E. coli* O157, there are roughly 2,000 clinical, food, or environmental *E. coli* O157 isolates belonging to 339 different clusters based on whole genome sequencing data (available from NCBI Pathogen Detection database, www.ncbi.nlm.nih.gov/pathogens/). Besides O157, over 50 other STEC serogroups are associated with human illness (mostly members of serogroups O26, O45, O103, O111, O121, and O145) making the range of pathogenic *E. coli* that require targeting by the food industry highly diverse [14].

The principal determinant of a phage’s infectivity is the binding specificity of its receptor binding proteins (RBPs), i.e., tail fibers and tailspikes (TSPs) [15]. Generally, TSPs are short spikes with enzymatic activity towards a saccharidic structure, while tail fibers are long fibrous proteins that only bind receptors as they lack enzymatic activity [16]. Phage RBPs have evolved to recognize a variety of structures on the surfaces of their bacterial hosts, including protein complexes (e.g., porins and the flagella) and saccharidic components such as teichoic acids, lipopolysaccharides (LPS), and capsular polysaccharides [17], [18], [19]. The inherent specificity of RBPs for a given species has led to their implementation in bacterial diagnostics, such as recently developed assays for *Listeria* [20]*, Salmonella* [21], [22]*, Pseudomonas* [23]*,* and *Yersinia* [24] detection. In addition, the ability of certain phage TSPs to enzymatically degrade the protective capsules of certain pathogens, e.g., *E. coli* [25]*, Klebsiella pneumoniae* [19], [26], and *Acinetobacter baumannii* [27], [28]*,* has led to their investigation as anti-virulence agents to target multidrug resistant bacteria and re-sensitize them to antibiotics.

The Gram-negative cell’s outer leaflet is composed almost exclusively of LPS. LPS consists of a membrane-bound lipid A, a core oligosaccharide, and a repetitive glycan polymer called the O-antigen. Variations in the O-polysaccharide repeat has given rise to over 170 unique O-antigens in *E. coli*, of which O157 is best known owing to its association with foodborne illnesses [29]. The majority of O-polysaccharide repeats in *E. coli* contain a backbone of four residues with a single residue side-branch, although the backbone can consist of two to six residues and be linear or branched. The O-antigens of the seven major STECs consist of three (O26, O45, O111, O145), four (O121, O157), or five (O103) residue repeats, can be branched (O111), and even sialylated (O145), demonstrating the wide variety of O-antigen structures across these important serogroups [30]. As one of the most abundant structures on the cell surface, the LPS is one of the most common receptors used by phages [18]. For example, tail fibers of *E. coli* phage T4 bind to the LPS core during host adsorption [31], whereas TSPs of *E. coli* phages G7C [32], HK620 [33] and CBA120 [34] actively degrade (or deacetylate in the case of G7C [32]) different O-antigen serotypes as they navigate the phage particle towards the bacterial cell surface prior to infection [35]. In general, TSP-carrying phages that recognize the O-antigen have narrow host ranges, as the previously described variation among *E. coli* O-antigens means that a single TSP will recognize one substrate or possibly a few closely related O-antigen structures [16]. As a compromise, various phages feature multiple TSPs or tail fibers to broaden their host ranges. For example, *E. coli* phage CBA120 features four TSPs that confer specificity towards different *E. coli* and *Salmonella* O-antigens [34], [36], [37], [38], whereas phages SP6 [39] and K1-5 [40] feature two rotatable TSPs to facilitate alternative host recognition. Bacterial resistance to phages can quickly develop during the application of phages, e.g., through phenotypic variation of bacterial subpopulations or receptor mutations [41]. As such, phage-based products are often composed of different phages that target unrelated receptors to draw selective pressure away from individual receptors and therefore reduce the likelihood that the target bacteria develop resistance.

Recently, we described the isolation and genomic characterization of two phages EP75 and EP335, which have broad and complementary specificity to *E. coli* O157 strains [43], [42] and have been developed as a cocktail that can significantly reduce *E. coli* O157 on beef when compared to chemical preservatives (e.g., lactic acid or peroxyacetic acid) [44]. To better understand the mechanisms governing the broad host ranges observed for these phages – especially toward O157 strains – we here focused on characterizing the structures and functions of their individual RBP complexes.

## 2 Materials and methods

### 2.1 Bacterial strains, bacteriophages, and culture conditions

*E. coli* and *Salmonella* strains were obtained from various culture collections as indicated in Table 1. Bacterial cultures were cultivated overnight in LB medium at 30 °C and 150 rpm agitation. Isolation and sequencing of phages EP75 and EP335 was described previously [42] with genomes available from GenBank under accession numbers MG748547 (vB_EcoM-EP75) and MG748548 (vB_EcoP-EP335).

**Table 1:** **Overview of phage EP335 and EP75 infectivity and RBP activity against *E. coli* and *Salmonella*.** Strain sources: 1, STEC center, Michigan state University, USA; 2, National Reference Centre for Enteropathogenic Bacteria and Listeria (NENT), University of Zürich, Switzerland; 3, Prof. Dr. Richard Calendar (University of California, Berkley, USA); 4, Public Health of England; 5, University of Würzburg, Germany. Phage activity: +, infected (plaque formation); (+), zones of turbidity (phage activity) but no visible plaque formation; -, no infection. Protein activity: ++, strong binding; +, weak binding; -, no binding; nt, not tested; *, TSP-treatment of cells reduced EP75 adsorption and infection (shown in Fig. 3, Fig. 4).

**Bacteria/serotype**	**Designation**	**Source**	**EP335 infection**	**GFP-gp13 binding**	**EP75 infection**	**EP75 TSP enzymatic activity (halo assay)**				Phage inhibition*
						**TSP4 (gp169)**	**TSP3 (gp169.1)**	**TSP2 (gp168)**	**TSP1 (gp167)**	
*E. coli* O157	TW01286	1	**+**	**++**	**+**	**–**	**–**	**+**	**–**	TSP2
*E. coli* O157	396	2	**+**	**++**	**+**	**–**	**–**	**+**	**–**	TSP2
*E. coli* O157	999/1	2	**+**	**++**	**+**	**–**	**–**	**+**	**–**	TSP2
*E. coli* O157	777/1	2	**(+)**	**++**	**+**	**–**	**–**	**+**	**–**	nt
*E. coli* O26	TW04584	1	**+**	**+**	**–**	**–**	**–**	**–**	**–**	nt
*E. coli* O26	TW04588	1	**+**	**+**	**–**	**–**	**–**	**–**	**–**	nt
*E. coli* O88	ECOR34	1	**+**	**–**	**–**	**–**	**–**	**–**	**–**	nt
*E. coli* O157	264	2	**(+)**	**–**	**–**	**–**	**–**	**–**	**–**	nt
*E. coli* K-12-derivative	C600	3	**(+)**	**–**	**–**	**–**	**–**	**–**	**–**	nt
*E. coli* O157	TW04583	1	**–**	**–**	**–**	**–**	**–**	**–**	**–**	nt
*E. coli* O26	NCTC08960	4	**–**	**–**	**–**	**–**	**–**	**–**	**–**	nt
*E. coli* O7	ECOR12	1	**–**	**–**	**–**	**–**	**–**	**–**	**–**	nt
*S.* Enteritidis H (O9)	Se1	5	**–**	**–**	**+**	**–**	**+**	**–**	**–**	TSP3
*S.* Enteritidis C (O9)	Se13	5	**–**	**–**	**+**	**–**	**+**	**–**	**–**	TSP3
*S.* Enteritidis I (O9)	Se2	5	**–**	nt	**+**	**–**	**+**	**–**	**–**	TSP3
*S.* Enteritidis D (O9)	Se26	5	**–**	nt	**+**	**–**	**+**	**–**	**–**	TSP3
*S.* Panama (O9)	Se22	5	**–**	nt	**+**	**–**	**+**	**–**	**–**	nt
*S.* Javiana (O9)	Se61	2	**–**	nt	**+**	**–**	**+**	**–**	**–**	nt
*S.* Typhimurium (O4)	Se5	5	**–**	nt	**+**	**–**	**+**	**–**	**–**	nt
*S.* Derby (O4)	Se46	2	**–**	nt	**+**	**–**	**+**	**–**	**–**	nt
*S.* Derby (O4)	Se45	2	**–**	nt	**+**	**–**	**+**	**–**	**–**	nt
*S.* Braenderup (O7)	Se32	5	**–**	nt	**+**	**–**	**+**	**–**	**–**	nt
*E. coli* O18A	DSM10809	[61]	nt	nt	**+**	**–**	**–**	**–**	**+**	TSP1
*E. coli* O18A1	TD2158	[61]	nt	nt	**–**	**–**	**–**	**–**	**–**	nt
*E. coli* O18B	DSM10837	[61]	nt	nt	**–**	**–**	**–**	**–**	**–**	nt
*E. coli* O18B1	DSM10922	[61]	nt	nt	**–**	**–**	**–**	**–**	**–**	nt

### 2.2 Protein expression and purification

A pQE30-based plasmid featuring N-terminal His- and GFP-tags [45] was used to express all RBP constructs. Cloning was performed by Gibson assembly (NEBuilder® HiFi DNA Assembly, New England Biolabs) using phage genomic DNA as a template and oligonucleotides listed in Supplementary Table 1. Plasmid sequences (Supplementary Table 1) were confirmed by Sanger sequencing (Microsynth AG, Switzerland) before transformation into XL1-Blue MRF’ cells for protein expression. For protein expression, cells were grown in LB media containing 100 μg/ml ampicillin until log-phase growth (OD_600_ of 0.6), when expression was induced with 0.5 mM isopropyl-D-thiogalactopyranoside (IPTG; Thermo Scientific, USA) for 16 h at 20 °C with agitation. Cells were harvested by centrifugation (5,500 × g, 10 min). Cells were suspended in phosphate buffered saline + 0.1% Tween-20, pH 8.0 (PBS-T), containing 5 mM imidazole, cooled to 4 °C, and lysed using a Stansted pressure cell homogenizer (Stansted Fluid Power, UK). Purification was performed by gravity flow immobilized metal affinity chromatography (IMAC) with low-density Ni-NTA resin (Agarose Bead Technology, USA). A solution of 0.1% PBS-T + 5 mM imidazole was used as a wash buffer after which 0.1% PBS-T, pH 8.0 + 250 mM imidazole was used for elution. Proteins were dialyzed into 25 mM Tris, pH 7.4 and stored at 4 °C. Proteins were analyzed by SDS-PAGE (Criterion TGX stain-free gel) using PageRuler Unstained Protein Ladder (Thermo Scientific™) as a marker and imaged by UV or stained with InstantBlue® (Expedeon) using a Gel Doc XR + Imaging system (BioRad).

### 2.3 Fluorescence assays

Overnight cultures of individual strains were spun down (5,000 × g, 5 min), resuspended to an OD_600_ of 1.0 in PBS-T, and transferred in 500 µl aliquots into 2 ml microcentrifuge tubes. 50 µg of GFP-tagged RBP was added to the cells and incubated for 90 min on an overhead rotator. Cells were spun down and washed once with 1 ml PBS-T and resuspended in 200 µl PBS-T. 150 µl of the cell suspension was pipetted into a well of a black, flat bottom 96-well microplate (Greiner Bio-One, Austria) and the fluorescence intensity of bound GFP-RBP was measured at ambient temperature using a POLARStar Omega spectrophotometer (BMG Labtech, Germany) at 485 nm excitation, 520 nm emission with (1000 x) fixed gain. Fluorescence binding was performed in triplicate with mean (raw fluorescence) ± standard deviation. For fluorescence microscopy, 4 μl of the cell suspension was imaged using a confocal inverted microscope (Leica TCS SPE) equipped with an ACS APO 63×/1.30 oil CS lens objective with excitation at 488 nm and emissions collected with a PMT detector in the detection range of 510 to 550 nm. Transmitted-light microscopy images were obtained with the differential interference contrast mode. Images were acquired with a Leica DFC 365 FX digital camera controlled with the LAS AF software. Fiji v2.0.0 (ImageJ software) was used to generate final images.

### 2.4 Tailspike halo assay

Bacterial lawns were prepared for each test strain by mixing 200 µl of overnight bacterial cultures with 5 ml soft agar (4 mg/ml agar) poured onto a LB agar plate (12 mg/ml agar) and left to solidify for 30 min. 10 µl of EP75 TSPs 1–4, GFP or BSA controls at 2 mg/ml were spotted onto the lawn and incubated for 16 h at room temperature, 30 °C, or 37 °C. Active TSPs produced translucent and circular haloes on target bacterial lawns. Plates were imaged with an iPhone XR 12-megapixel camera under a lightbox with contrast and brightness adjustments applied to whole images to improve halo visualization.

### 2.5 Bacteriophage enumeration

The double layer agar method [46] was used to enumerate phages EP75 and EP335. Briefly, phage preparations of EP75 or EP335 were serially diluted in 1x SM buffer (50 mM Tris, pH7.5, 100 mM NaCl, 8 mM MgSO_4_), after which an appropriate volume was added to 4 ml Yeast Glucose agar (25 g/L yeast, 1% glucose, 0.3% agar) containing 100 µl of an *E. coli* NCTC13128 overnight culture. Subsequently, the 4 ml LB top agar was poured over an LB agar bottom plate (1.5% agar) and allowed to solidify at room temperature. The titration plates were then incubated overnight at 30 °C followed by plaque enumeration.

### 2.6 Bacteriophage adsorption inhibition assay

To determine the inhibitory effect of EP75 TSP enzymatic activity of the *E. coli* or *Salmonella* cell surfaces on phage adsorption, overnight cultures of *E. coli* or *Salmonella* were diluted to an OD_600_ of 0.5 in 200 µl LB medium. TSPs or GFP (0.025 mg/ml final concentration) or no protein were then added to the cells and incubated for 10 min at room temperature. The cells were collected by centrifugation (10,000 × *g*, 2 min) and resuspended in 200 µl LB medium. Approximately 300 plaque forming units (PFU) of EP75 was added to the resuspended pellets followed by an incubation of 10 min at room temperature. The solution was centrifuged (10,000 × *g*, 2 min) and 100 µl of supernatant (containing non-bound phages) was mixed with 4 ml Yeast Glucose agar containing 100 µl *E. coli* NCTC13128 and poured on top of an LB agar plate. Plates were incubated at 30 °C overnight followed by PFU enumeration (total non-bound phages; PFU_supernatant_). A sample containing 300 PFU of EP75 and no bacteria or protein was treated the same and used as input control (PFU_input_). Relative phage adsorption was calculated as (PFU_input_ - PFU_supernatant_)/PFU_input_.

### 2.7 Phage infection inhibition assay

To determine the inhibitory effect of EP75 TSP enzymatic activity on *E. coli* or *Salmonella* cells on phage efficiency of plating a phage infection inhibition assay was performed. For *E. coli* strains, a single colony was inoculated in LB broth and incubated at 37 °C at 150 rpm. After reaching an OD_600_ of 0.3, aliquots of 100 µl of the culture were immediately incubated on ice to stop growth. TSPs or GFP (0.2 mg/ml final concentration) or no protein was added to the aliquots and incubated for 20 min at 37 °C. For *Salmonella* strains, an overnight culture was diluted to OD_600_ of 0.3 in LB medium and divided in 100 µl aliquots at room temperature. TSPs or GFP (0.2 mg/ml final concentration) or no protein was added to the aliquots and incubated for 20 min at room temperature. Approximately 100 PFU of EP75 were added to individual aliquots (*Salmonella* and *E. coli*), mixed with 4 ml Yeast Glucose agar and poured on top of an LB agar plate. Plates were incubated at 30 °C overnight followed by PFU enumeration and determination of the efficiency of plating (EOP): PFU_treatment_/PFU_no protein_.

### 2.8 Mass spectrometry

Protein masses were identified using Liquid Chromatography-Electrospray Ionization-Mass Spectrometry (LC-ESI-MS) at the Functional Genomics Center Zürich, Switzerland (www.FGCZ.ch) using standard protocols. In brief, samples were diluted 2-fold with 1% trifluoroacetic acid and transferred to autosampler vials for LC/MS. 10 μl of sample was injected into an ACQUITY UPLC@ BioResolve-RP-mAb 2.7μ 2.1x150 450 A (Waters, USA) column. For separation and elution on an Acquity UPLC station (Waters, USA), a gradient buffer A (0.1% formic acid in water)/ buffer B (0.1% formic acid in acetonitrile) at a flow rate of 200 µl/min at 500C over 25 min was applied. Analysis was performed on a Synapt G2 mass spectrometer (Waters, UK) directly coupled with the UPLC station. Mass spectra were acquired in the positive-ion mode by scanning an *m*/*z* range from 100 to 4000 Da with a scan duration of 1 s and an interscan delay of 0.1 s. The spray voltage was set to 3 kV, the cone voltage to 50 V, and source temperature 80 °C. The data were recorded with the MassLynx 4.2 Software (Waters, UK). For single peaks, the recorded *m*/*z* data were then deconvoluted into mass spectra by applying the maximum entropy algorithm MaxEnt1 (MaxLynx) with a resolution of the output mass 0.5 Da/channel and Uniform Gaussian Damage Model at the half height of 0.5 Da.

### 2.9 Statistical analysis

Data presented in all graphs were obtained from three independent experiments and shown as the mean ± standard deviation. Statistical significance was calculated by two-way ANOVA with *post hoc* Tukey’s multiple comparison test using GraphPad Prism (version 8.2.0).

### 2.10 Bioinformatics analysis

BLASTp sequence comparisons were performed using the NCBI website platform with default parameters. BLASTn sequence alignments were performed with 30% minimal identity on 100 base pair (bp) minimum alignments using in-house, python-based software.

## 3 Results and discussion

### 3.1 Characterization of phage EP335 tail fibers gp12 and gp13

**Fig. 1:**
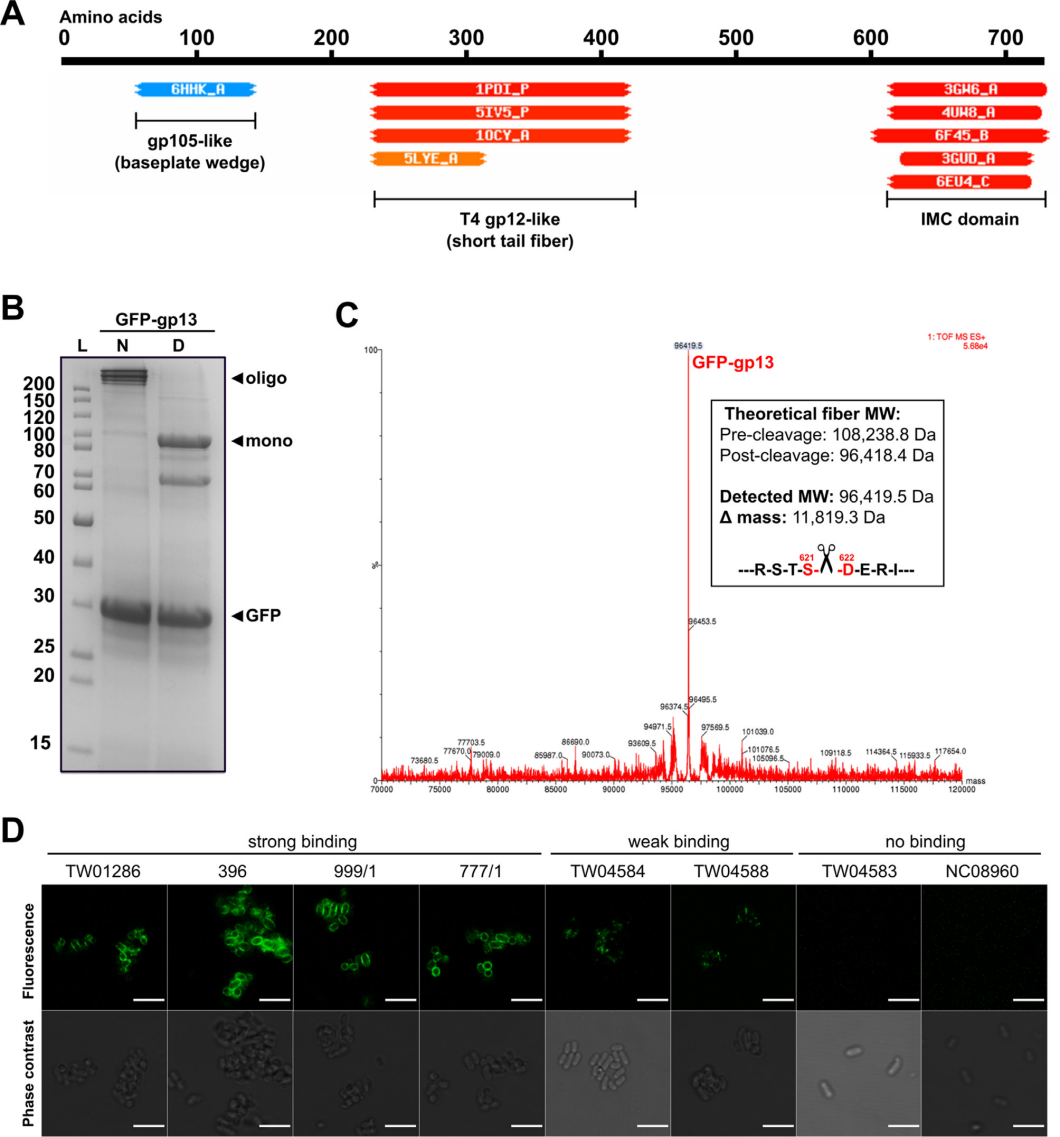
**Identification and characterization of the tail fiber (gp13) of phage EP335. A**) HHpred analysis [47] identified regions of similarity with other phage RBP structures. **B**) SDS-PAGE of Ni-NTA-purified GFP-gp13. Under native conditions (N; non-boiled), GFP-gp13 formed SDS-resistant oligomers, as observed with other phage RBPs. Heat denaturing (D; 96 °C, 8 mins) caused dissociation into the monomeric form (96.4 kDa; after intermolecular chaperone cleavage). In both samples a GFP contaminant band (~30 kDa) was observed. **C**) ESI-LC-MS spectra of GFP-gp13 detected a MW of 96,419.5 Da, which corresponds with auto-proteolysis of the C-terminal IMC domain after Ser621. **D**) Fluorescence and phase contrast images of GFP-gp13 cell binding to different *E. coli* strains. Cell binding correlated with fluorescence spectroscopy in **Supplementary**Fig. 2. Scale bars represent 5 µm.

Whole genome sequencing previously identified phage EP335 (GenBank: MG748548) as a member of the *Kuravirus* genus (previously *Phieco32virus*) within the *Podoviridae* family [42], with high sequence identity to other genus members such as *E. coli* phages KBNP1711 (96.7% identity, 88% coverage; GenBank: KF981730), NJ01 (85.5% identity, 69% coverage; GenBank: JX867715) and the type phage phiEco32 (85.5% identity, 67% coverage; GenBank: EU330206). To explain the broad and specific binding range of EP335 towards O157 strains (76/88 strains tested [43]), we characterized two putative RBPs, gp12 and gp13, identified within its genome. HHpred analysis [47] predicted structural similarity between regions of gp13 and other known phage RBPs (Fig. 1**A**). The central segment of gp13 (Phe236-Ala422) was predicted to resemble the homotrimeric tip of the *E. coli* phage T4 short tail fiber (STF; gp12). In addition, the C-terminal 114 residues of gp13 were predicted to form an intramolecular chaperone domain (IMC), which assist with the folding and maturation of phage RBPs before autoproteolytic removal, as described for the long tail fiber of phage S16 (PDB ID: 6F45) [48], the TSP of phage K1F (PDB ID: 3GW6) [49], and the *Bacillus* phage GA-1 neck appendage (PDB ID: 3GUD) [49]. HHpred did not identify any similar structures to gp12, and Pfam [50] only identified a domain of unknown function (DUF3251) within the gp12 N-terminus (Leu90-Ser168). The tail fibers of *E. coli* siphoviruses DT57C and DT571/2 (LtfA and LtfB) and podovirus phiEco32 (gp14 and gp15) have been determined to form a branched fiber structure whereby LtfB/gp15 connects to LtfA/gp14, which then attaches the dual fiber complex via its N-terminus to the phage tail apparatus [51]. Interestingly, the tail fibers of EP335 and phiEco32 share high sequence identity (gp12 to gp14, 67%; gp13 to gp15, 61.5%), suggesting a similar branched tail fiber network forms on the EP335 baseplate.

Is common for phage RBPs [52], [53], [54], SDS-PAGE revealed SDS resistant oligomer formation for GFP-gp13, which were reduced to the monomeric state after heat denaturation (Fig. 1**B**). As shown in Fig. 1**C**, LC-ESI-MS analysis of GFP-gp13 shows a reduction in the molecular weight by 11.8 kDa, corresponding to cleavage between Ser621 and Asp622, which confirmed our previous identification of a self-cleaved C-terminal IMC domain. Similar to other IMC-containing homotrimers, cleavage occurs after a highly conserved serine residue (Ser621) [49], [55]. Using fluorescence microscopy and spectroscopy, recombinant GFP-tagged constructs of both fibers were produced and their ability to interact with *E. coli* hosts was assessed. GFP-gp13 bound strongly and evenly to the surfaces of *E. coli* O157 strains susceptible to phage EP335 infection, i.e., enabled plaque formation (Table 1 and Fig. 1**D**). GFP-gp13 also bound to phage-susceptible O26 strains TW04584 and TW04588; however, the level of binding was visibly weaker than that against the O157 strains as observed by fluorescence microscopy (Fig. 1**D**), with the fluorescent signal too weak to detect by fluorescence spectroscopy (Supplementary Fig. 2). Binding was also observed for GFP-gp13 against *E. coli* strain 777/1 (O157), which was prone to EP335 activity (turbid zones produced by phage EP335 spots) but lacked discernable plaque formation. However, *E. coli* strains 264 (O157) and the C600 rough strain (lacking O-antigen) that are prone to phage activity but not plaque formation, were not bound by GFP-gp13. Finally, *E. coli* ECOR34, which features a branched O88 antigen, was the only strain susceptible to phage infection that was not bound by GFP-gp13. Unfortunately, no binding was observed for GFP-gp12 against any strains tested (Supplementary Fig. 1). Based off high sequence identity (gp12, 91%; gp13, 63%) with homologous fibers from a related Kuravirus EcoN5 (MN715356) propagated on serotype O6 *E. coli* ATCC 25922, we additionally tested the binding of gp12 and gp13 to three O6 strains (ECOR-10, −11, and −56); however, no binding was observed for either protein. While gp13 functions as an RBP binding to O157 and O26 *E. coli* strains, there are various hypotheses that could explain the lack of binding by GFP-gp12: (i) attachment of GFP to gp12 renders the protein non-functional; (ii) gp12 requires an additional intermolecular chaperone, similar to other phage RBPs [52], [56], [57], [58]; (iii) the affinity of gp12 to its receptor is too weak to maintain interaction for the duration of the assay; or (iv) gp12 does not possess receptor binding functionality. Further investigations into the role of gp12 are necessary, in addition to exploring the gp12 and gp13 dual fiber network proposed here based on homology to the phiEco32 fibers [51].

### 3.2 Characterization of the tailspike network of phage EP75

**Fig. 2:**
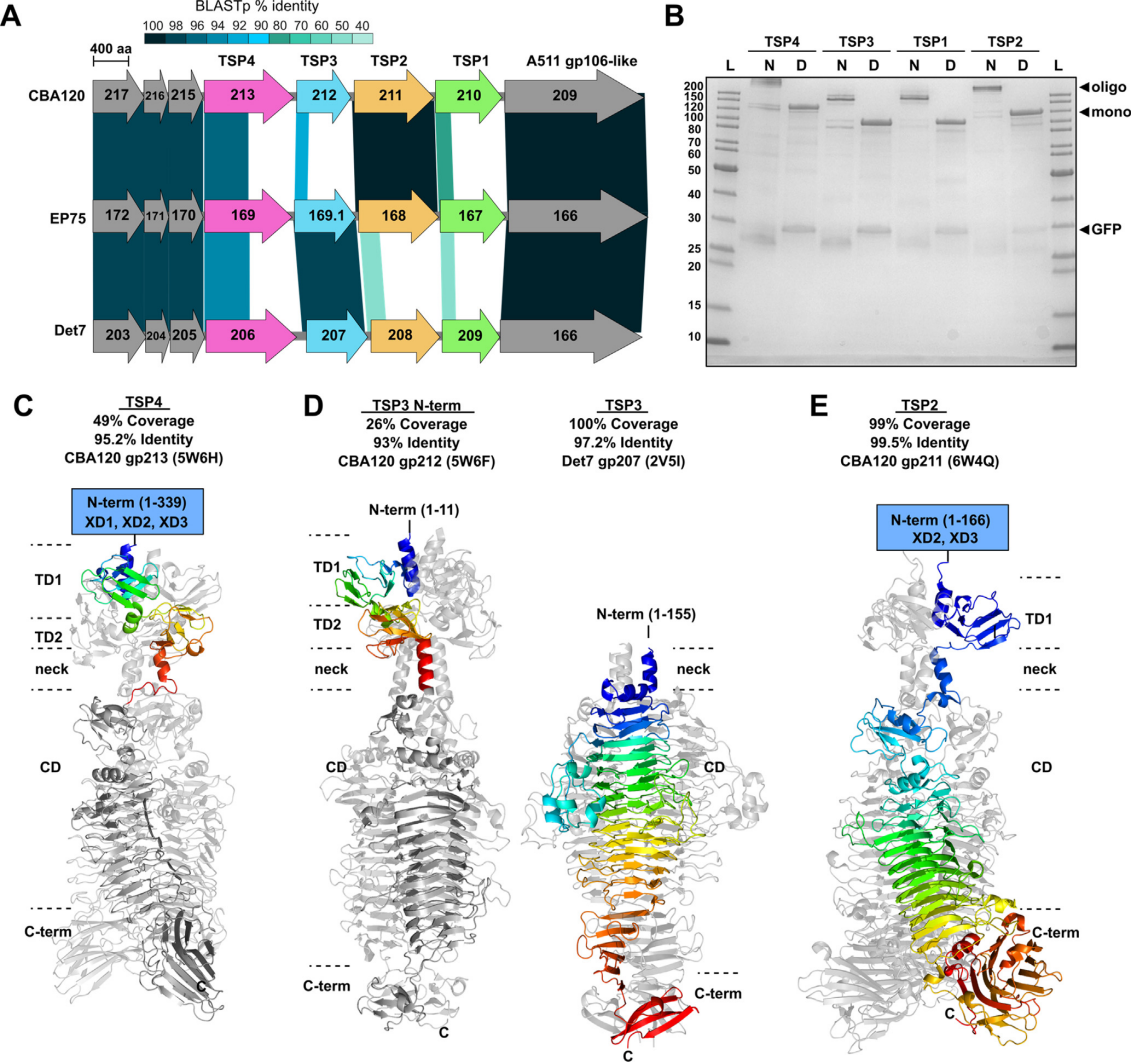
**Identification and structural analysis of EP75 tailspikes. A**) BLASTp analysis of EP75 structural modules and TSPs with phages CBA120 and Det7. Darker alignments indicate a higher percentage identity between sequences. Gaps indicate no sequence homology, although synteny is still preserved. **B**) SDS-PAGE of GFP-tagged TSPs after Ni-NTA purification. All four TSPs formed SDS-resistant oligomers under native conditions (N; non-boiled), similar to GFP-gp13 shown in Fig. 1. All four TSPs separated into their monomeric form after heat denaturation (D; 96 °C, 8 min). A GFP contaminant band (~30 kDa) was present in all four purifications. **C-E**) Ribbon representations of CBA120 and Det7 TSP crystal structures with high sequence and structural similarity to EP75 TSP4 and TSP2, and N- and C-terminal regions of TSP3. For each structure, a single chain is rainbow colored (from N-terminus, blue, to C-terminus, red) to highlight the segment with sequence similarity to the EP75 TSPs, with dark grey indicating no similarity. Individual domains are identified: XD1-3, gp10-like attachment domains [34]; TD1/2, tandem domains 1 and 2; neck domain; CD, catalytic β-helical domain; C-terminal domain. Panels C, D, and E were generated using PyMOL Molecular Graphics System, Version 1.4 Schrödinger, LLC. (For interpretation of the references to colour in this figure legend, the reader is referred to the web version of this article.)

Whole genome sequencing previously identified phage EP75 as a member of the *Kuttervirus* genus within the *Ackermannviridae* family [28], with high sequence identity to other members such as *E. coli* phages PhaxI (96.6% identity, 91% coverage; GenBank: JN673056) and type phage CBA120 (93% identity, 90% coverage; GenBank: JN593240), as well as *Salmonella* phages such as Det7 (93% identity, 89% coverage; GenBank: KP797973). We aimed to understand the broad and cross-genus host range (73/88 *E. coli* O157 and 12/43 *Salmonella* strains tested [43]) of EP75 through characterization of its RBPs and their respective host recognition functions. Alignment of the structural genes of EP75 with related phages CBA120 and Det7 revealed conservation of synteny and regions of high sequence identity for all four TSPs (Fig. 2**A and** Supplementary Fig. 2). Crystal structures of all four CBA120 TSPs have been resolved [34], [36], [37], [38] (Fig. 2, Fig. 4), as well as the organization of its TSP network [34]. As is common for TSPs, the C-terminal segment of all four TSPs forms a variable length, trimeric right-handed parallel β-helix with distinct enzymatic activity: TSP2, TSP3 and TSP4 specifically target O157, O77, and O78 O-antigens, respectively, whereas TSP1 targets the LPS of *Salmonella* Minnesota [34], [36], [37], [38]. The N-terminal regions of the four CBA120 TSPs also differ. In brief, the N-terminus of TSP4 contains three T4 gp10-like attachment domains that connect to the baseplate and N-terminal regions of TSP1 and TSP2 [34]. TSP2 also contains two N-terminal gp10-like domains for connecting to the N-terminal regions of TSP4 and TSP3 and completing the TSP network [34]. The high sequence similarity between the N-terminal regions of EP75 and CBA120 TSPs (Fig. 2**A**) indicates conservation of this RBP network.

Here, GFP-tagged constructs of the four EP75 TSPs were produced (Fig. 2**B**) to explore their individual receptor binding and enzymatic properties against different *E. coli* and *Salmonella* hosts (Table 1). No binding was observed by any of TSPs; however, as shown for CBA120 TSP2 against *E. coli* [34], interaction by these TSPs is transient and undetectable by fluorescence microscopy. Nevertheless, spotting of TSP1 (gp167), TSP2 (gp168), or TSP3 (gp169.1) generated turbid haloes indicative of enzymatic activity when spotted onto bacterial lawns of *E. coli* O18A, *E. coli* O157, or *Salmonella* O4, O7 and O9 strains, respectively (Table 1 **&** Supplementary Fig. 3). No halo formation was observed for TSP4 (gp169) against any strains tested.

### 3.3 TSP2 and TSP3 analysis

**Fig. 3:**
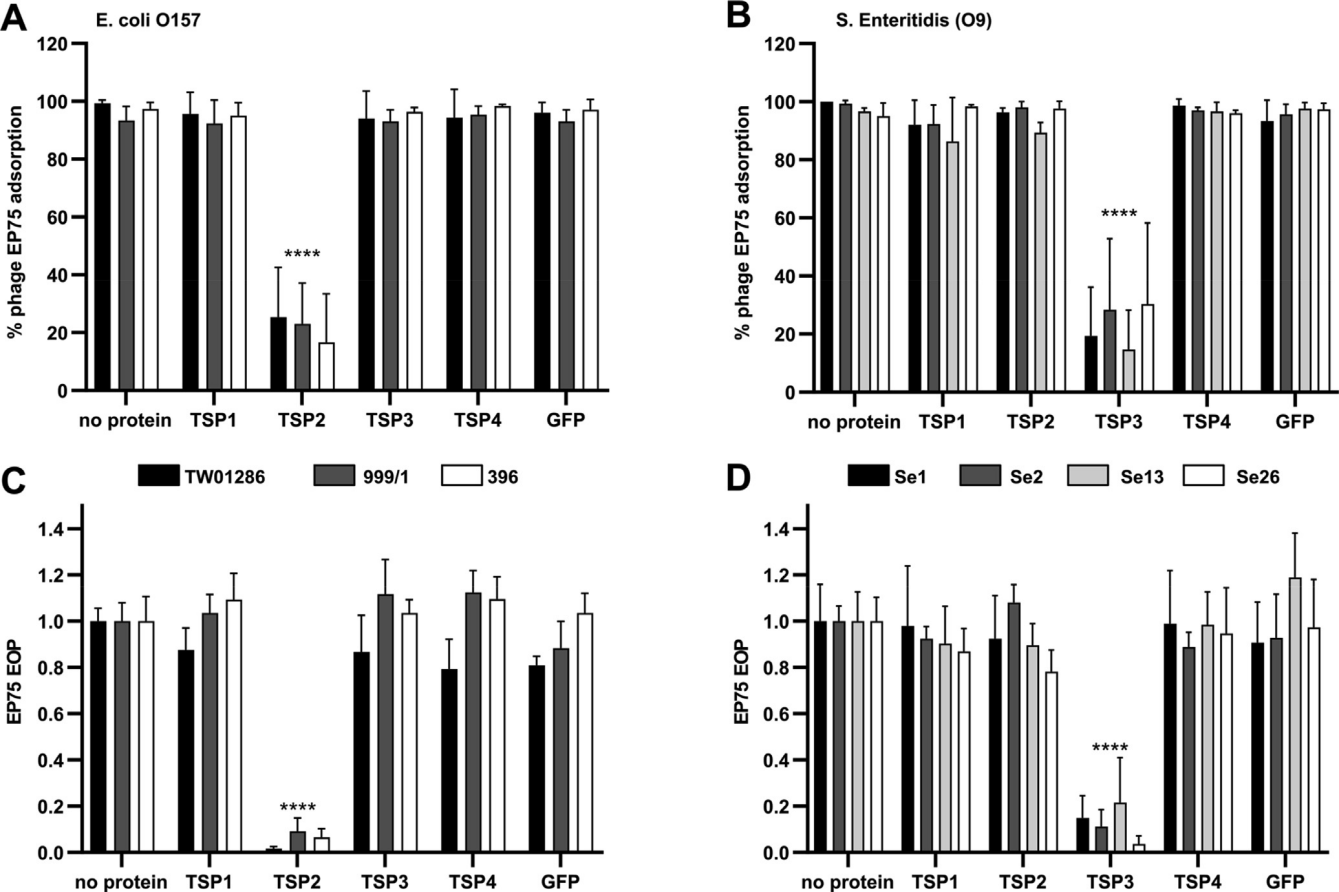
**Inhibition of adsorption and infection of EP75 to *E. coli* O157 and *Salmonella* O9 strains by TSP2 and TSP3.** EP75 phage particles added to *E. coli* (**A**) or *Salmonella* (**B**) pre-treated with TSP2 or TSP3, respectively, had significant reduction in their cell binding ability. No effect was observed when treated with TSP1, TSP4 or GFP. The efficiency of plating (EOP) of EP75 against *E. coli* (**C**) or *Salmonella* (**D**) strains was also significantly reduced in the presence of TSP2 or TSP3. Data presented as mean ± SD. Two-way ANOVA showed significant difference for TSP2 or TSP3 treatments only (****, P < 0.0001), with no significance observed among the other treatments.

Given the 99.5% sequence identity (99% coverage) of the EP75 TSP2 and its CBA120 counterpart (Fig. 2**E**), including conservation of all active site residues [34], [36], it was not surprising to see activity toward the *E. coli* O157 strains tested. Interestingly, the EP75 TSP2 was not active against phage-resistant O157 strains (Table 1), suggesting possible modification to the O-antigens of these strains (not identified by serotyping) or other extracellular barriers that affected TSP2 activity. The N-terminal 160 residues of the CBA120 TSP3 (gp212) forms two tandem domains that connect the tailspike to the N-terminus of TSP2 during assembly of the TSP network [34]. TSP3 of CBA120 shares 93% identity with the first 150 residues of its EP75 counterpart, meaning the N-terminal region and the two tandem domains are maintained for the EP75 TSP3 (Fig. 2**D**). There is no similarity between the C-terminal domain of EP75 TSP3 and the TSP3 of CBA120, which instead presented 97.2% sequence similarity (100% coverage) with the O-antigen degrading TSP (gp207) of *Salmonella* phage Det7 (Fig. 2**D**), including conservation of all active site residues [54]. The EP75 TSP3 was active against various *Salmonella* serovars that feature O4 or O9 antigens and are susceptible to EP75 infection such as *S.* Enteritidis (O9), *S.* Panama (O9)*, S.* Typhimurium (O4), and *S.* Derby (O4) (Table 1). The O4 and O9 activity of TSP3 correlated with the host range of phage Det7 [59] and interaction observed for Det7 TSP (gp207) to purified *S.* Typhimurium O-antigen [54]. We also observed TSP3 activity against *S.* Braenderup (O7), which is also susceptible to EP75 infection [43]. The effect of the enzymatic activity of TSP2 and TSP3 on phage EP75 adsorption and infectivity (i.e., efficiency of plating; EOP) was further assessed. As expected, incubation of *E. coli* O157 strains with TSP2 significantly reduced the number of phages that could adsorb to the cells (<25% average pulled down compared to > 93% pulldown with no protein) due to binding and degrading of its O-antigen receptor (Fig. 3**A**). Similarly, the EOP of EP75 (i.e., number of plaques formed compared to no protein control) was significantly reduced when O157 strains were incubated with TSP2, again due to the enzymatic removal of the O-antigen receptor required for infection of these strains (Fig. 3**C**). No significant difference in EOP or phage adsorption could be observed when other TSPs or GFP were added as they all produced similar results as the protein-free control. Treatment of *Salmonella* strains with TSP3 followed a similar trend and produced a significant reduction in the adsorption and infectivity of phage EP75 towards the *Salmonella* strains tested (Fig. 3 **B&D).** Again, no significant difference in EP75 adsorption or infectivity was observed when other TSPs or GFP control were added to *Salmonella*.

### 3.4 TSP1 analysis

**Fig. 4:**
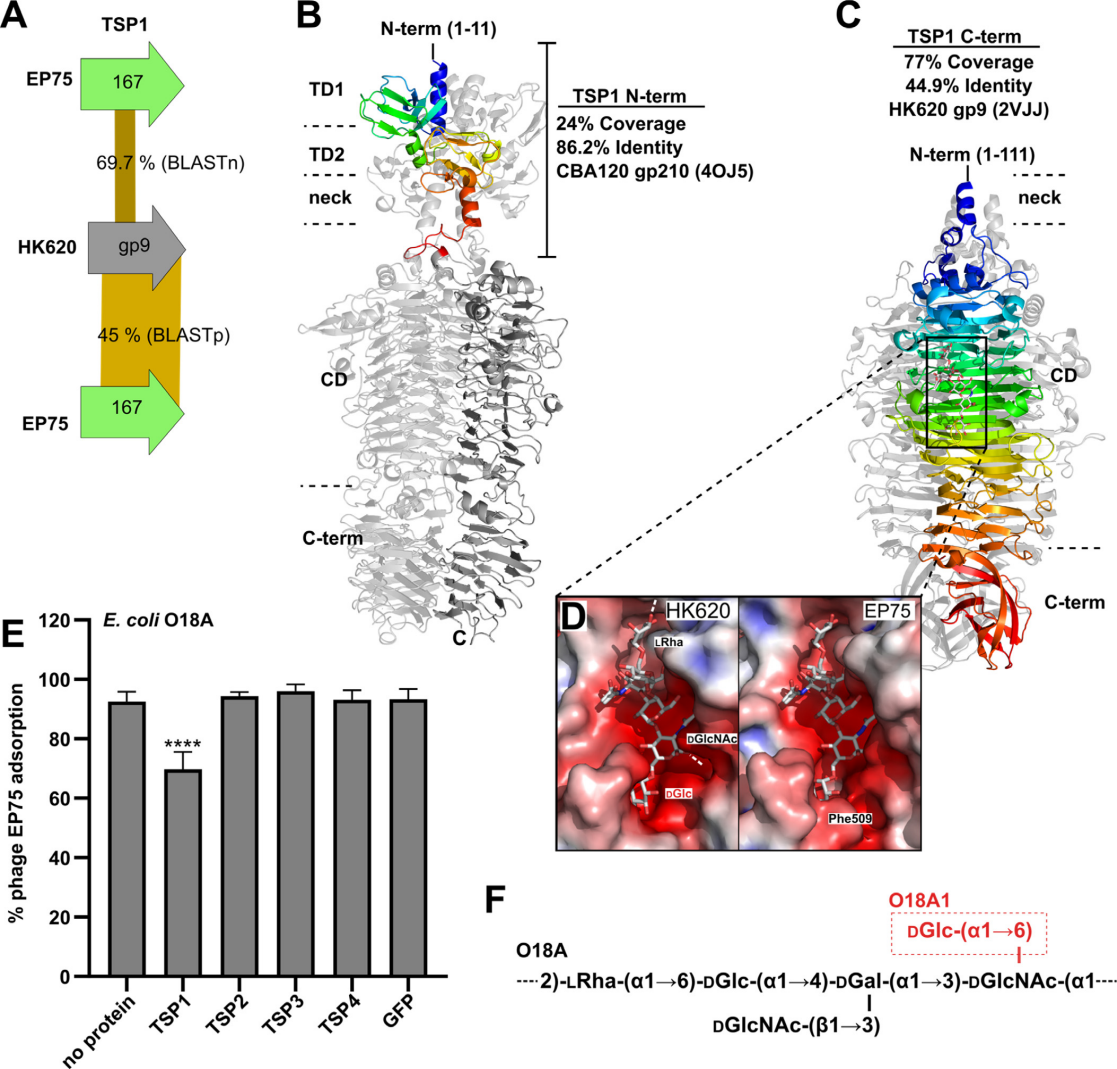
**Characterization of the O18A-specific TSP1 of phage EP75. A)** Percentage identity for BLASTn (top) and BLASTp (bottom) analyses of TSP1 and the TSP (gp9) of phage HK620. **B & C**) Ribbon representations of CBA120 and HK620 TSP crystal structures with sequence and structural similarity to the N-terminal and C-terminal regions of TSP1. For each structure, a single chain is rainbow colored (from N-terminus, blue, to C-terminus, red) to represent regions of sequence similarity. Dark grey in panel B indicates no sequence similarity. **D)** Highlights the molecular surfaces of the HK620 TSP (PDB ID: 2VJJ [33]) and TSP1 (generated by SWISS-MODEL [63] using 2VJJ as a template) catalytic sites bound to the O18A1 hexasaccharide repeat unit. Molecular surfaces are colored according to electrostatic surface potential using APBS (red, negative charged; white, neutral charged; and blue, positive charged regions (±5 kT/e) [67]. The end pocket occupied by the branched glucose of the O18A1 hexasaccharide is smaller for TSP1, which could explain the observed specificity of TSP1 toward only the O18A antigen. Individual domains are also identified: TD1/2, tandem domains 1 and 2; neck domain; CD, catalytic β-helical domain; C-terminal domain. **E**) EP75 phage particles added to *E. coli* DSM10809 cells pre-treated with TSP1 had significant reduction in their cell binding ability. Data presented as mean ± SD. Two-way ANOVA showed significant difference for TSP1 treatment (****, P < 0.0001), with no significance observed among the other treatments. **F**) The O–antigen repeat unit of type O18A and O18A1 [62]. Panels B, C, and D were generated using PyMOL Molecular Graphics System, Version 1.4 Schrödinger, LLC. (For interpretation of the references to colour in this figure legend, the reader is referred to the web version of this article.)

TSP1 shares high similarity with only the N-terminal region of its CBA120 TSP1 counterpart (86.2% identity, 24% coverage). HHpred analysis revealed the C-terminus of TSP1 (Ser158 to end) shares high structural similarity and 45% sequence identity with the well-characterized TSP (gp9) of *Salmonella* podovirus HK620 (NC_002730.1) [33] (Fig. 4). HK620 gp9 is an *endo*-*N*-acetylglucosaminidase TSP that specifically targets and degrades *E. coli* O18A1 LPS [33], [60], [61]. The catalytic residues of HK620 gp9 (E417_EP75_/E372_HK620_ and D384_EP75_/D339_HK620_) [33] are maintained within a central conserved region between the two TSPs, suggesting TSP1 functions also as an endoglycosidase, potentially targeting *Salmonella* O18 serogroup LPS. Phage EP75 could infect the O18A strain DSM10809, with TSP1 also producing clear haloes against this strain; however, no activity could be observed for phage EP75 or TSP1 against the O18A1, O18B, or O18B1 strains tested (Table 1). As confirmation of its O18A-specific activity, treatment of *E. coli* DSM10809 with TSP1 significantly reduced the ability of phage EP75 to bind to the bacteria with no difference in adsorption observed when treated with other TSPs or a GFP control (Fig. 4**D**). Interestingly, the only difference between the O18A1 and O18A LPS is the former has a branched glucose (Glu) linked to the terminal *N*-Acetylglucosamine (GlcNAc) of the repeating unit (Fig. 4**E**) [62] suggesting that subtle differences between the binding cavities of the two TSPs are responsible for the different O18 serogroup specificities. A structure of TSP1 was generated via homology modeling using SWISS-MODEL [63] and the crystal structure of HK620 TSP bound to a O18A1 hexasaccharide repeat unit as a template (PDB ID: 2VJJ [33]). While the overall shape, charge, and positioning of catalytic residues were maintained between the two TSPs, the pocket occupied by the branched glucose (specific to O18A1) appeared constricted by a Gly to Phe switch in the TSP1 model (Fig. 4**C Inset**), which potentially explains the lack of activity against the branched O18A1 LPS.

### 3.5 TSP4 analysis

As no halo formation or phage inhibition could be observed for TSP4 against any of the strains, we attempted to determine its putative enzymatic activity based on sequence and structural similarity to other characterized phage TSPs. The first half of TSP4 shares 95.2% and 93.7% sequence identity with its CBA120 and Det7 counterparts, respectively, suggesting conservation of the N-terminal attachment domains necessary for construction of the three TSP networks for connecting to the phage baseplate (Fig. 2**A)**. BLASTp analysis demonstrated > 95% sequence identity (100% coverage) with other *Salmonella* phage TSPs from the *Kuttervirus* genus, e.g., phages ST-W77 (NC_049378.1), moki (NC_049506.1), and SJ_3 (NC_024122.1), which has been used to treat *Salmonella* infections in pigs [64], [65]; however, individual receptors of these related TSPs have not been determined. Despite their low sequence similarity (<20%), HHpred predicted TSP4 (probability, 100%; E value, 2.9 × 10^-41^) as having the same structure as CBA120 TSP4 (grey region, Fig. 2**C**). Nevertheless, while the CBA120 TSP4 targets and degrades the O78 antigen of *E. coli* [34], EP75 does not infect, and neither phage EP75 or its TSP4 demonstrate activity against, O78 strains (e.g., *E. coli* ECOR-71). Given that all *E. coli* and *Salmonella* serotypes within the current host range of EP75 could be accounted for by corresponding activity of TSP1, TSP2, or TSP3, we expect TSP4 to target a different *Salmonella* or *E. coli* serotype or even another species, which requires further investigation.

## 4 Conclusion

By combining structure and sequence analyses with enzymatic and cell binding experimentation we gained a deeper understanding of the host range determining characteristics of the EP75 and EP335 RBPs. While the target of TSP4 remains elusive, the cross-genus infectivity of EP75 toward certain *E. coli* and *Salmonella* serotypes can now be explained by the individual activities of TSP1 (*E. coli* O18A), TSP2 (*E. coli* O157), and TSP3 (*Salmonella* O4, O9, O7). Overall, the TSPs of EP75 provide excellent examples of the modular nature of phage genomes and the exchange observed between functional modules, i.e., between C-terminal enzymatic domains of phage TSPs during evolution [15], [16], [66]. While we could not discern the target or function of EP335 gp12, based on sequence similarities with *E. coli* siphoviruses DT57C and DT571/2 and podovirus phiEco32 [51], this putative tail fiber is predicted to form a dual-branched tail fiber network with gp13, whose binding to O157 and O26 strains was demonstrated here (Fig. 1**)**. Given the infectivity of EP335 towards these two serotypes, their unbranched O-antigen structures are expected to be the primary receptor used by the phage. However, this does not remove the possibility that other surface structures are also used by EP335 for adsorption or infection, especially as the two RBPs could still recognize different surface structures.

Finally, it is interesting to note that O157 strains 264 and TW04583, which are not infected by either phage, also are not recognized by their respective RBPs (TSP2 of EP75 and gp13 of EP335). Similarly, non-infected O26 strain NCTC08960 is also not recognized by EP335 gp13. Potentially, modification to the O-antigens (not identified by serotyping) or other extracellular barriers exist for these and other non-infected O157 or O26 strains and requires further investigation. Overall, this study demonstrates how structure- and sequence-based characterization of phage RBPs can be used to provide important information on the different receptors used by phages, which could aid the selection of phages with broader infectivity for implementation in phage-based antibacterial and therapeutic products in the future.

## Author contributions

Conceptualization MD and JTvM; Investigation MD, SW, JTvM, LZ, RGS, CM; Project administration MD; Resources MJL; Supervision MD, JTvM; Validation MD; Visualization MD; Roles/Writing - original draft MD; Writing - review & editing MD, JvTM, MJL, SW, RGS, LZ.

## CRediT authorship contribution statement

**Sander Witte:** Investigation, Writing - review & editing. **Léa V. Zinsli:** Investigation, Writing - review & editing. **Rafael Gonzalez-Serrano:** Investigation, Writing - review & editing. **Cassandra I. Matter:** Investigation. **Martin J. Loessner:** Resources, Writing - review & editing. **Joël T. van Mierlo:** Conceptualization, Investigation, Supervision, Writing - review & editing. **Matthew Dunne:** Conceptualization, Investigation, Project administration, Supervision, Validation, Visualization, Writing - original draft, Writing - review & editing.

## Declaration of Competing Interest

The authors declare that they have no known competing financial interests or personal relationships that could have appeared to influence the work reported in this paper.
