# Supplementary material for: Structural and functional characterization of the receptor binding proteins of Escherichia coli O157 phages EP75 and EP335
Source: Comput Struct Biotechnol J. 2021 Jun 4;19:3416–26. doi: 10.1016/j.csbj.2021.06.001 (PMC8217332; doi:10.1016/j.csbj.2021.06.001)
Supplement: Supplementary data 1 [file mmc1.docx]

**Supplementary Information**

**Supplementary Table 1.** List of plasmids constructed for this study and corresponding primers used for cloning.

| **Plasmid** | **Other designation and features** | **Primer name** | **5' to 3' sequence** |
| --- | --- | --- | --- |
| pQE30-His-GFP (empty) | Ampr; IPTG‐induced; 6xHis- & GFP- tags. GFP control protein | General_Fw | GCC CTG GAA ATA CAG ATT CTC G |
|  |  | General_Bw | AAT TAG CTG AGC TTG GAC TCC T |
| pQE30-His-GFP-gp167 | GFP-tagged EP75 TSP4 | TSP4_Fw | CGAGAATCTGTATTTCCAGGGCATGAACGAAATGTTTAGTCAAGGT |
|  |  | TSP4_Bw | AGGAGTCCAAGCTCAGCTAATTTTATGTAAAGTTTTGCAAATACACATTA |
| pQE30-His-GFP-gp168 | GFP-tagged EP75 TSP3 | TSP3_Fw | CGAGAATCTGTATTTCCAGGGCATGGGGTATTTTCAAATGACTAGAAA |
|  |  | TSP3_Bw | AGGAGTCCAAGCTCAGCTAATTTTAAATAGAAGAATCCAATATACGGTAA |
| pQE30-His-GFP-gp169.1 | GFP-tagged EP75 TSP2 | TSP2_Fw | CGAGAATCTGTATTTCCAGGGCATGATTTCTCAATTCAATCAACCACGC |
|  |  | TSP2_Bw | AGGAGTCCAAGCTCAGCTAATTTTACACAGATAACTTCATACGTGTTT |
| pQE30-His-GFP-gp169 | GFP-tagged EP75 TSP1 | TSP1_Fw | CGAGAATCTGTATTTCCAGGGCATGGCCAACAAACCAACACA |
|  |  | TSP1_Bw | AGGAGTCCAAGCTCAGCTAATTTCAAATTGTCAATGTGTTGACTAAGGT |
| pQE30-His-GFP-gp12 | GFP-tagged EP335 gp12 fiber | gp12_Fw | CGAGAATCTGTATTTCCAGGGCATGATCGTTTATAATAACCAAGCACCT |
|  |  | gp12_Bw | AGGAGTCCAAGCTCAGCTAATTTTACTCGGTTGGCATCTCTACA |
| pQE30-His-GFP-gp13 | GFP-tagged EP335 gp13 fiber | gp13_Fw | CGAGAATCTGTATTTCCAGGGCATGTCTAGAGAATTAATGCCCAAAT |
|  |  | gp13_Bw | AGGAGTCCAAGCTCAGCTAATTTTACTTACTAGACTTAAGCTCTGACA |

**
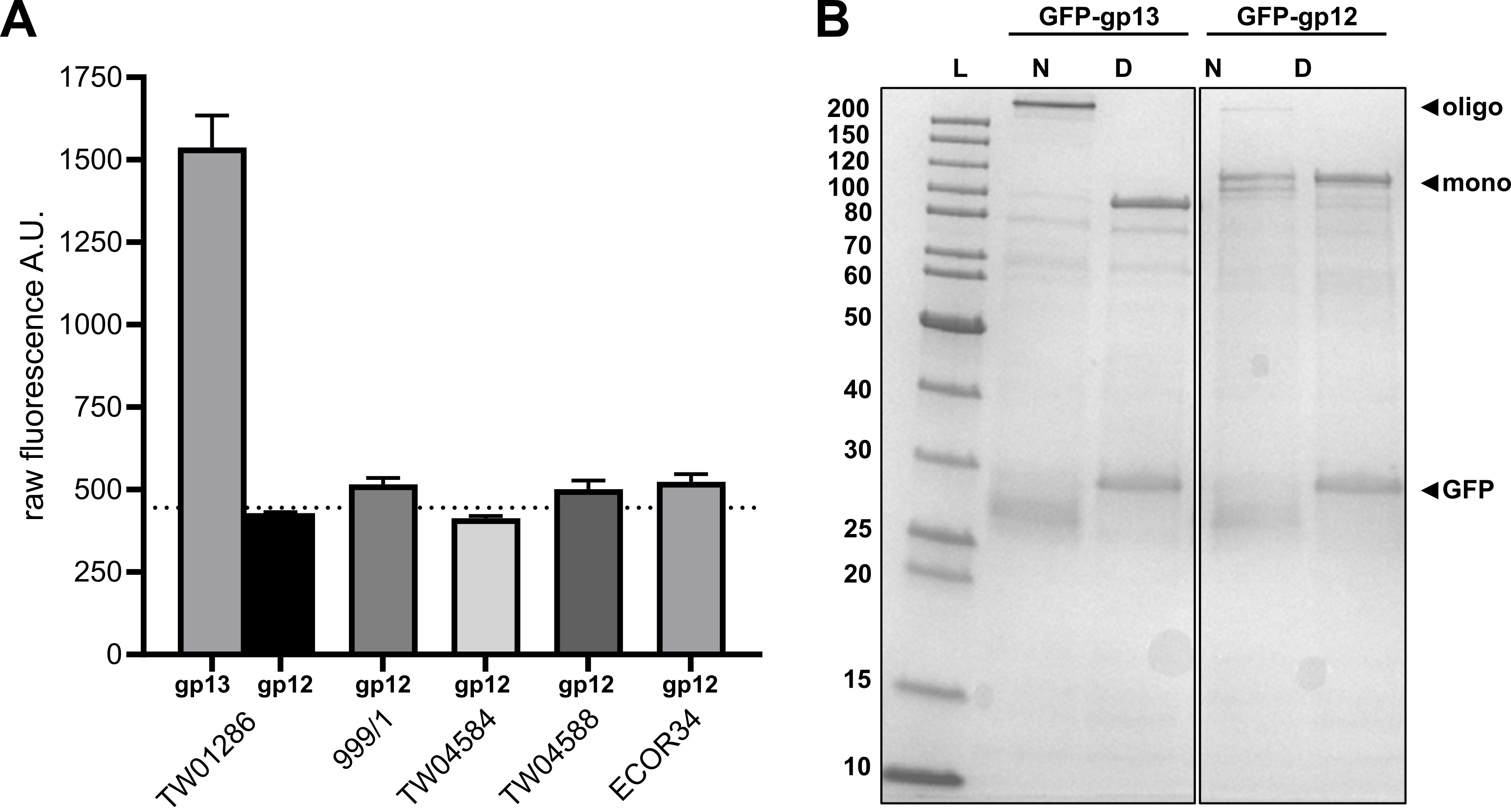
**

**Supplementary Figure 1. GFP-tagged gp12 from phage EP335 does not demonstrate cell binding. A)** GFP-gp12 did not demonstrate cell binding to any of the phage susceptible *E. coli* strains shown. GFP-gp13 decoration of *E. coli* TW01286 shown as representative positive binding. Dotted line indicates the average fluorescence observed for non-bound strains tested with GFP-gp13. **B)** SDS-PAGE of GFP-tagged gp12 and gp13 after Ni-NTA purification. N, non-boiled; D, heat denatured. Only GFP-gp13 showed clear separation into monomers after heat denaturation (D; 96°C, 8 min). A GFP contaminant band (~30 kDa) was present in both purifications.


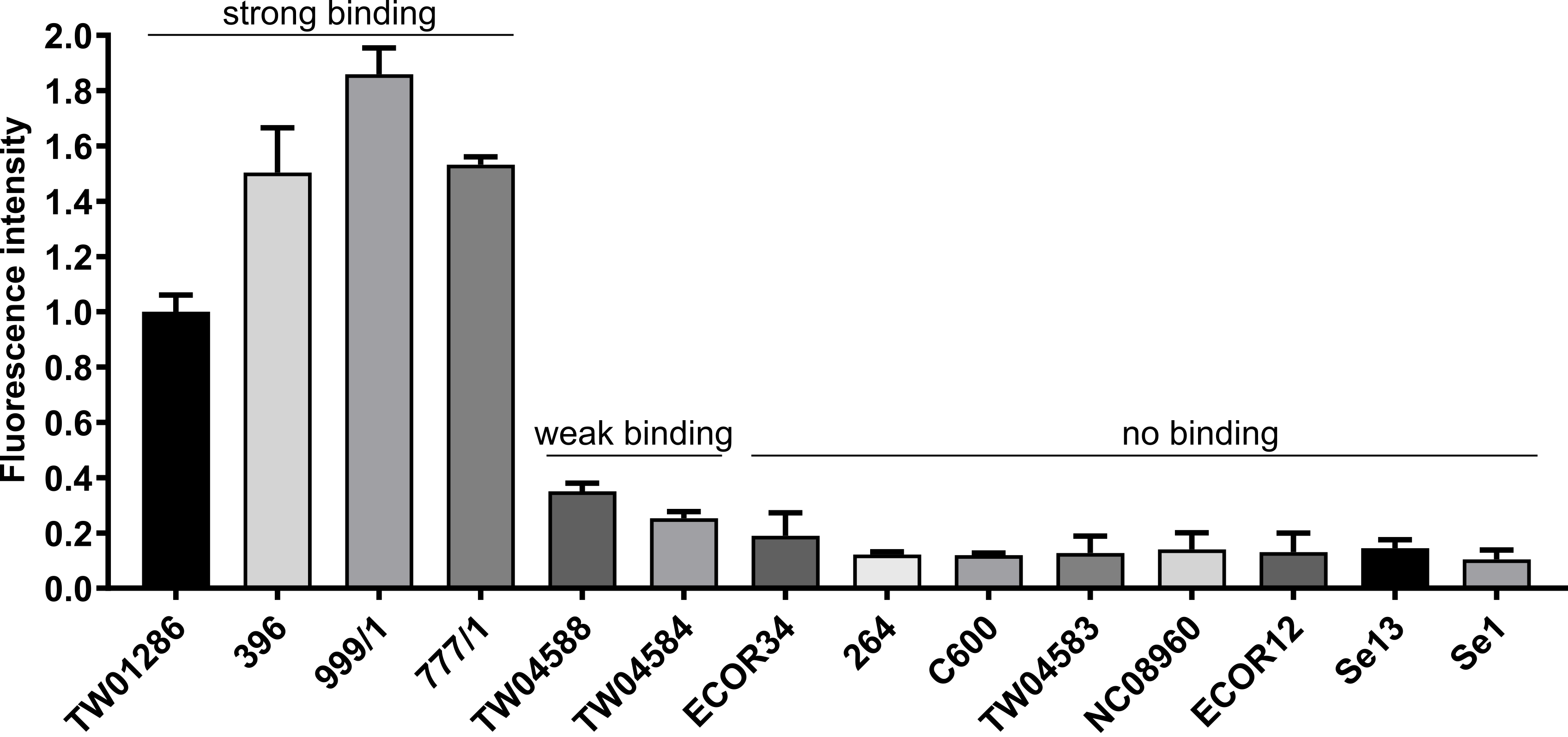


**Supplementary Figure 2.** Fluorescence spectrometry measurement of GFP-gp13 binding to *E. coli* O157 (TW01286, 396, 999/1, 777/1, TW04583, 264), O26 (TW04584, TW04588, NC08960) O88 (ECOR34), O7 (ECOR12), and K-12 derivative (C600) strains, and *S. Enteritidis* strains (Se13 and Se1). Fluorescence intensity (relative to propagation host TW01286) ± SD of triplicate measurements are shown. *, P ≤ 0.05; ns, no significance. Strong, weak, or no binding refers to observation of GFP-gp13 interaction by microscopy (shown in **Figure 1**).


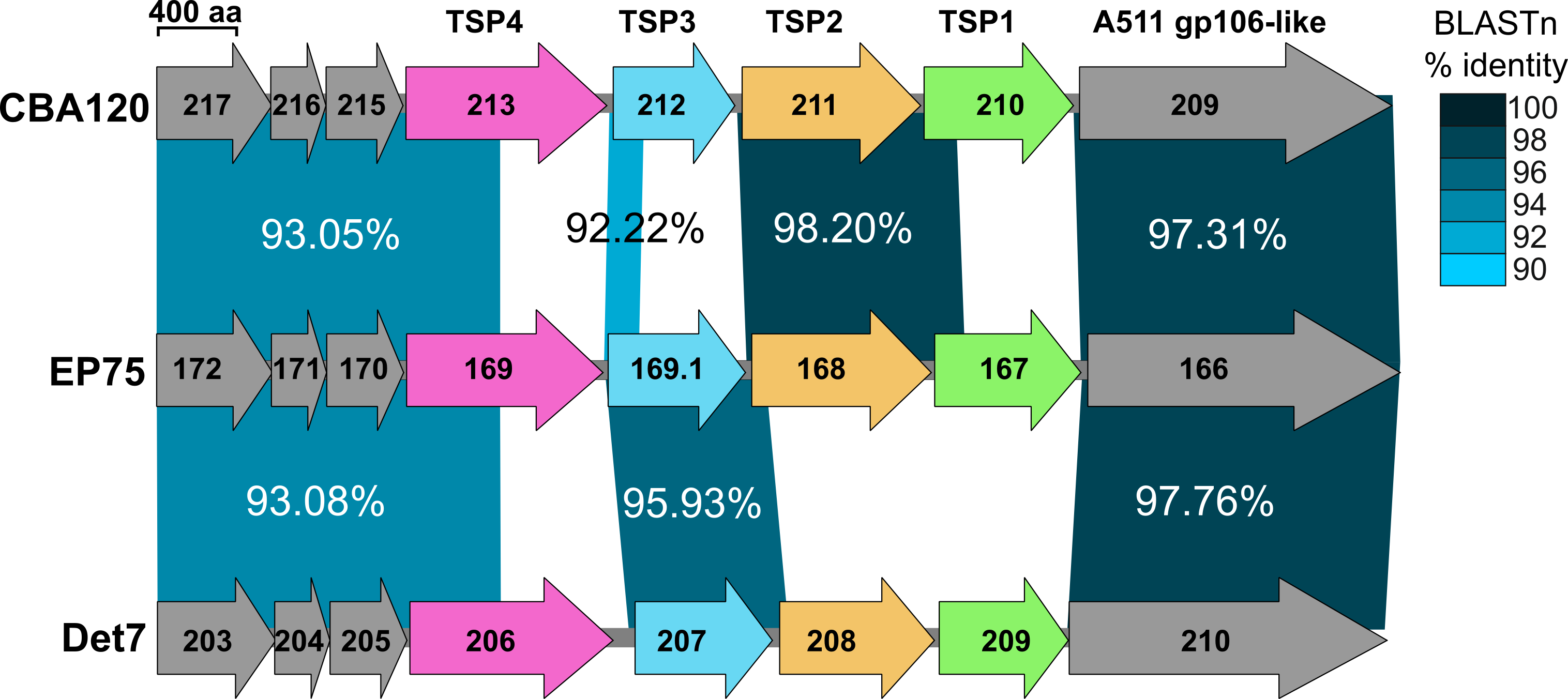


**Supplementary Figure 3. BLASTn analysis of EP75 structural modules and TSPs with phages CBA120 and Det7.** Alignment of the structural modules and TSPs of phages EP75, CBA120 and Det7 using BLASTn with 30% minimal identity on 100 base pair (bp) minimum alignments. Darker alignments indicate a higher percentage identity between sequences. Gaps indicate no sequence homology, although synteny is still preserved in these regions.


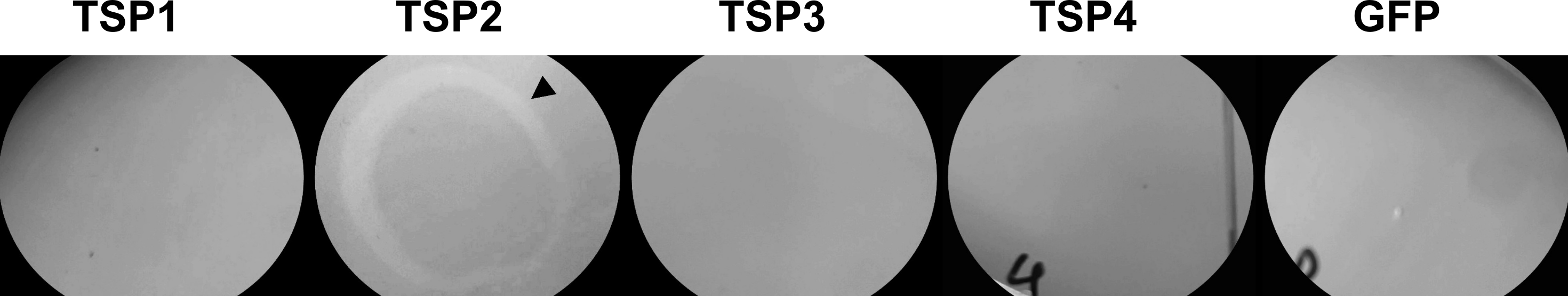


**Supplementary Figure 4. Representative image of halo formation.** Halo formed only for TSP2 enzymatic activity on *E. coli* O157 strain TW01286**.** Black arrow indicates the halo periphery. No halo or activity was observed for other TSPs or GFP control.
